# Cardiotoxicity of combined pegylated liposomal doxorubicin and bevacizumab therapy: a propensity-matched cohort study and disproportionality analysis

**DOI:** 10.1186/s40959-025-00351-4

**Published:** 2025-07-02

**Authors:** Christopher W. Hoeger, Arrush Choudhary, Andrea Nathalie Rosas Diaz, Theresa Pinto, Sarah Smalec, Charles Doladille, Rishi Wadhera, Meghan Shea, Sumanth Khadke, Joe-Elie Salem, Sarju Ganatra, Aarti Asnani

**Affiliations:** 1 Division of Cardiovascular Medicine, Beth Israel Deaconess Medical Center, Harvard Medical School, Boston, MA USA; 2 Montefiore-Einstein Center for Heart and Vascular Care, Montefiore Medical Center, Albert Einstein College of Medicine, Bronx, NY United States of America; 3 Department of Medicine, Beth Israel Deaconess Medical Center, Harvard Medical School, Boston, MA USA; 4 Department of Pharmacy, Beth Israel Deaconess Medical Center, Harvard Medical School, Boston, MA USA; 5 Normandie University, UNICAEN, INSERM U1086 ANTICIPE, Caen, France; 6 PICARO Cardio-Oncology Program, Department of Pharmacology, CHU de Caen-Normandie, Caen, France; 7 Division of Medical Oncology, Beth Israel Deaconess Medical Center, Harvard Medical School, Boston, MA USA; 8 Department of Cardiovascular Medicine, Lahey Hospital & Medical Center, Burlington, MA USA; 9 Assistance Publique Hôpitaux de Paris, Pitie-Salpêtrière Hospital, INSERm, Sorbonne University, Paris, CIC-1901 France; 10 3 Blackfan Circle, CLS-911, Boston, MA 02115 USA

**Keywords:** Pegylated liposomal doxorubicin, Bevacizumab, Cardiotoxicity, Heart failure, Ovarian cancer, Pharmacovigilance, Disproportionality analysis

## Abstract

**Background:**

Pegylated liposomal doxorubicin (PLD) and bevacizumab are commonly used to treat platinum-resistant ovarian cancer. While both agents are associated with cardiovascular toxicities, their combined impact on cardiotoxicity in real-world settings is not well defined. This study investigates whether co-administration of PLD and bevacizumab increases the risk of cardiovascular adverse events compared to PLD alone.

**Methods:**

A retrospective cohort study was conducted using the TriNetX Analytics Network Database. Patients treated with PLD and bevacizumab were matched 1:1 to those receiving PLD alone using propensity score matching. Cardiovascular outcomes, including heart failure, cardiomyopathy, hypertension, and venous thromboembolism, were assessed over two years. Replication was performed using VigiBase, the World Health Organization’s global adverse drug reaction database, through disproportionality analysis.

**Results:**

Among 1,194 matched patients in each group, combination therapy was associated with increased risks of heart failure or cardiomyopathy (OR 1.42, 95% CI 1.07–1.88, *P* = 0.015), hypertension (OR 1.80, 95% CI 1.36–2.38, *P* < 0.001), venous thromboembolism (OR 1.23, 95% CI 1.02–1.49, *P* = 0.029), and all-cause mortality (OR 1.26, 95% CI 1.07–1.48, *P* = 0.005). VigiBase analysis confirmed disproportionate reporting of hypertension (ROR 6.05, 95% CI 4.61–7.94), heart failure (ROR 1.75, 95% CI 1.23–2.47), and pericardial disorders (ROR 3.67, 95% CI 1.61–8.38) in patients receiving combination therapy.

**Conclusions:**

Combined PLD and bevacizumab therapy was associated with increased risk of cardiotoxicity compared to PLD alone. These findings emphasize the need for proactive cardiovascular monitoring in patients undergoing this combination treatment. Prospective studies are warranted to further elucidate the underlying mechanisms and to refine clinical management strategies.

**Supplementary Information:**

The online version contains supplementary material available at 10.1186/s40959-025-00351-4.

## Background

Pegylated liposomal doxorubicin (PLD) and bevacizumab are cornerstones of the contemporary treatment of platinum-resistant epithelial ovarian cancer. Both traditional, non-liposomal anthracyclines and bevacizumab are associated with a variety of cardiovascular toxicities when given in isolation, including cardiomyopathy, heart failure, arrhythmia, and myocardial infarction [1, 2]. Studies of chemotherapy regimens that combine non-liposomal doxorubicin and bevacizumab show a substantial risk of heart failure, resulting in limited use of this combination in clinical practice [3, 4]. PLD is thought to reduce the risk of cardiotoxicity as compared to traditional anthracyclines through decreased myocardial drug accumulation [5, 6]. In the clinical trial setting, combination treatment with PLD and bevacizumab has been associated with an increased risk of hypertension, but this regimen has not been reported to confer an excess risk of other types of cardiotoxicity [7]. The incidence of cardiotoxicity of combined therapy in the real-world setting is not known. We therefore sought to determine whether the combined use of PLD and bevacizumab conferred an increased risk of cardiotoxicity in two large, retrospective patient cohorts.

## Methods

### Study population and design

#### TriNetX database

The risk of major cardiac adverse events (MACE) with combined PLD and bevacizumab, as compared to PLD alone, was assessed via a matched multicenter cohort study using the TriNetX Analytics Network Database-Research Network. TriNetX is a large-scale multicenter federated health research network that aggregates anonymized electronic health record data from participating academic medical centers, specialty physician practices and community health centers, with the research network containing data on more than 88 million patients from 59 healthcare organizations [8]. 

The TriNetX database was queried and data curated on September 28th, 2022, to generate a cohort of patients of at least 18 years of age who received treatment with PLD and bevacizumab, as well as PLD alone, using validated diagnostic, procedural, and laboratory codes. We identified a cohort of patients receiving PLD alone with similar characteristics to those of patients receiving both PLD and bevacizumab using propensity score matching. Patients entered the study when starting cancer treatment and were followed for 2 years.

The matched cohorts were compared for rates of new cardiomyopathy or heart failure, acute myocardial infarction, atrial fibrillation or flutter, venous thromboembolism, peripheral artery disease, new diagnosis of hypertension, or death of any cause.

#### VigiBase

The findings of the TriNetX cohort were then replicated through an analysis of cardiovascular adverse drug reactions (CV-ADRs) reported in VigiBase, the World Health Organization’s (WHO) international database of individual case safety reports from over 130 countries. These reports originate from different sources, including healthcare professionals, patients, and pharmaceutical companies via post-marketing notification.

VigiBase was queried for selected specific CV-ADRs among reports of adverse reactions to PLD according to a combination of Preferred Term levels of the Medical Dictionary for Regulatory Activities (MedDRA; version 25.1), as previously described, between inception (November 14, 1967) through September 1, 2022) [9]. To determine if combination use was associated with higher reporting of CV-ADRs, we assessed co-reporting of bevacizumab use within reports of PLD use. Reporting of the VigiBase data conforms to the READUS-PV statement for reporting of disproportionality analyses (supplemental Table 1) [10]. 

### Statistical analysis

Descriptive statistics are presented as frequency for categorical variables and mean and standard deviation for continuous variables. Baseline characteristics were compared between two groups using independent-sample t-tests for continuous variables and Chi-squared tests for categorial variables. To control baseline differences between the study cohorts, we performed propensity-score matching (PSM) using greedy nearest‐neighbor matching with a caliper of 0.1 times the pooled standard deviation of the linear propensity scores. The standardized mean difference between cohorts from the TriNetX database was calculated both before and after propensity matching. A standardized mean difference < 0.1 was considered an adequate balance for a variable between the treated and untreated groups. Survival analyses were performed by plotting Kaplan-Meier curves with log-rank tests and calculating hazard ratios via log-rank testing to compare cohorts. Statistical significance was set at a 2-sided P value less than 0.05.

The VigiBase cohort was assessed using disproportionality analysis (also known as case-non-case analysis), comparing the proportion of a selected specific CV-ADR reported for a combination of PLD plus bevacizumab with the proportion of the same CV-ADR for a control group of PLD without bevacizumab. The denominator was the total number of adverse drug reactions reported for each treatment. A proportion of CV-ADR greater in patients exposed to a specific drug (cases) than in patients not exposed to this drug (non-cases) was consistent with an association between the specific drug and the reaction. Disproportionality was calculated using a frequentist disproportionality estimate, given as the reporting odds ratio (ROR) and its 95% confidence interval. A reporting odds ratio with a lower 95% confidence bound > 1 was considered significant. Very rare events with fewer than 5 cases reported in either group were excluded from analysis.

Statistical analysis of the TriNetX data was completed using the TriNetX online platform using R for statistical computing, and the analysis of the VigiBase data was performed using the *vigicaen* package for R version 0.15.4 (R Foundation for Statistical Computing) [11].

## Results

### Sample characteristics of the TriNetX cohort

A total of 1,515 patients were identified in the TriNetX database who received combination therapy and 2,564 patients who received PLD alone (Table 1). After propensity matching, 1,194 patients remained in each group and were similar with respect to age, malignancy diagnosis, metastatic disease, cardiovascular comorbidities, and cardiovascular medications.

**Table 1:** Baseline characteristics of TriNetX cohort before and after propensity matching

Baseline characteristics	Before propensity matching			After propensity matching		
	PLD plus Bevacizumab	PLD without Bevacizumab	SD	PLD plus Bevacizumab	PLD without Bevacizumab	SD
	(***n* = 1**,**515)**	**(*n* = 2**,**564)**		**(** ***n*** ** = 1194)**	**(** ***n*** ** = 1194)**	
**Demographics**						
Mean Age (years)	64.1 ± 10.8	61.6 ± 13.9	0.202	64.7 ± 10.6	64.7 ± 11.4	0.002
Female	1,508 (99.5%)	2,161 (84.3%)	0.583	1,187 (99.4%)	1,189 (99.6%)	0.024
White	1,221 (80.6%)	1,844 (71.9%)	0.205	936 (78.4%)	935 (78.3%)	0.002
Black or African American	177 (11.7%)	484 (18.9%)	0.201	162 (13.6%)	161 (13.5%)	0.002
**Comorbidities**						
Malignant neoplasms of female genital organs	1442 (95.2%)	1184 (46.2%)	1.277	1121 (93.9%)	1121 (93.9%)	< 0.001
Metastatic disease	1185(78.2%)	1695 (66.1%)	0.273	889 (74.5%)	890 (74.5%)	0.002
Hypertension	789 (52.1%)	1129(44.0%)	0.162	606 (50.8%)	614 (51.4%)	0.013
Hyperlipidemia	542 (35.8%)	773 (30.1%)	0.120	419 (35.1%)	424 (35.5%)	0.009
Diabetes mellitus	261 (17.2%)	470 (18.3%)	0.029	218 (18.3%)	223 (18.7%)	0.011
Atrial fibrillation/flutter	91 (6.0%)	139 (5.4%)	0.025	69 (5.8%)	72(6%)	0.011
Heart Failure	80 (5.3%)	138 (5.4%)	0.005	53 (4.4%)	53 (4.4%)	< 0.001
Cardiomyopathy	53 (3.5%)	115 (4.5%)	0.050	39 (3.3%)	36 (3.0%)	0.014
Ischemic Heart Disease	199 (13.1%)	277 (10.8%)	0.072	139 (11.6%)	138 (11.6%)	0.003
History of prior PCI	10 (0.7%)	10 (0.4%)	0.037	10(0.8%)	10(0.8%)	< 0.001
Cerebral Infarction	79 (5.2%)	62 (2.4%)	0.146	28 (2.3%)	34 (2.8%)	0.032
Chronic Kidney disease	147 (9.7%)	229 (8.9%)	0.027	107 (9.0%)	110 (9.2%)	0.009
Chronic lower respiratory diseases	261(17.2%)	416(16.2%)	0.027	207(17.3%)	208 (17.4%)	0.002
**Medications**						
Aspirin	439 (29.0%)	727 (28.4%)	0.014	338 (28.3%)	324 (27.1%)	0.026
Beta Blockers	622 (41.1%)	944 (36.8%)	0.087	492 (41.2%)	498 (41.7%)	0.010
Statins	493 (32.5%)	785 (30.6%)	0.041	396 (33.2%)	400 (33.5%)	0.007
ACE inhibitors	350 (23.1%)	495 (19.3%)	0.093	251 (21.0%)	253 (21.2%)	0.004
Angiotensin II inhibitor	215 (14.2%)	293 (11.4%)	0.083	166 (13.9%)	157 (13.1%)	0.022
Calcium channel blockers	330 (21.8%)	500 (19.5%)	0.056	252 (21.1%)	252 (21.1%)	< 0.001

Values expressed as mean ± standard deviation. Proportions are given as percentages in parentheses. PLD—pegylated liposomal doxorubicin; SD—standard difference; PCI—percutaneous coronary intervention; ACE—angiotensin converting enzyme

### Outcomes of the TriNetX cohort

**Fig. 1:**
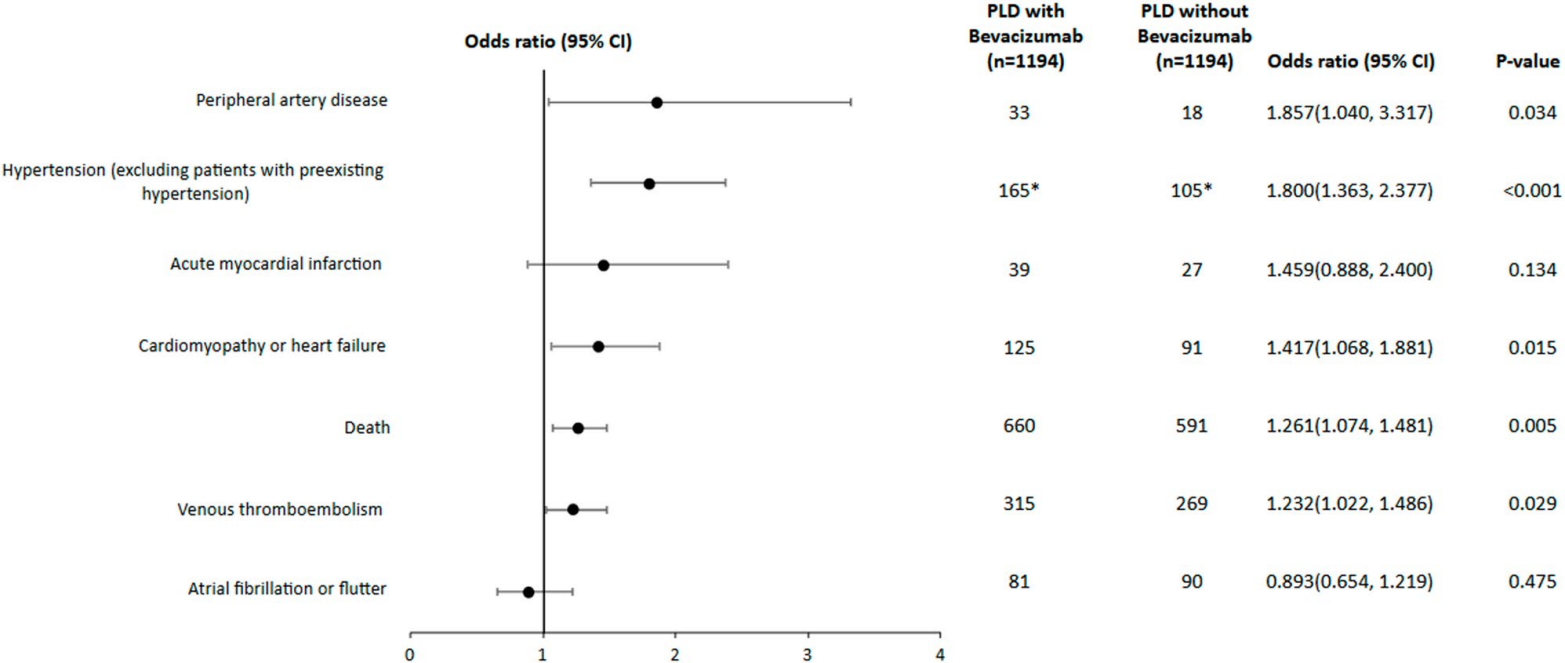
Cumulative risk of cardiovascular events for patients receiving PLD + bevacizumab as compared to a propensity-matched cohort receiving PLD alone in the TriNetX cohort. Patients with pre-existing hypertension were excluded from the analysis of the odds of hypertension. 578 patients were included in each group for the hypertension analysis. Odds ratios are shown with 95% confidence intervals

Co-treatment with PLD and bevacizumab was associated with increased risk of new heart failure or cardiomyopathy (Fig. 1, OR 1.417 95% CI 1.068–1.881, *P* = 0.015), venous thromboembolism (OR 1.232, 95% CI 1.022–1.486, *P* = 0.029), peripheral artery disease (1.857 95% CI 1.040–3.317, *P* = 0.034), hypertension (OR 1.800 95% CI 1.363–2.377, 95% CI < 0.001), and all-cause mortality (OR 1.261, 95% CI 1.074–1.481, *P* = 0.005) as compared to treatment with PLD alone in the matched cohorts. There was no significant difference in the risk of acute myocardial infarction or atrial fibrillation / flutter.

**Fig. 2:**
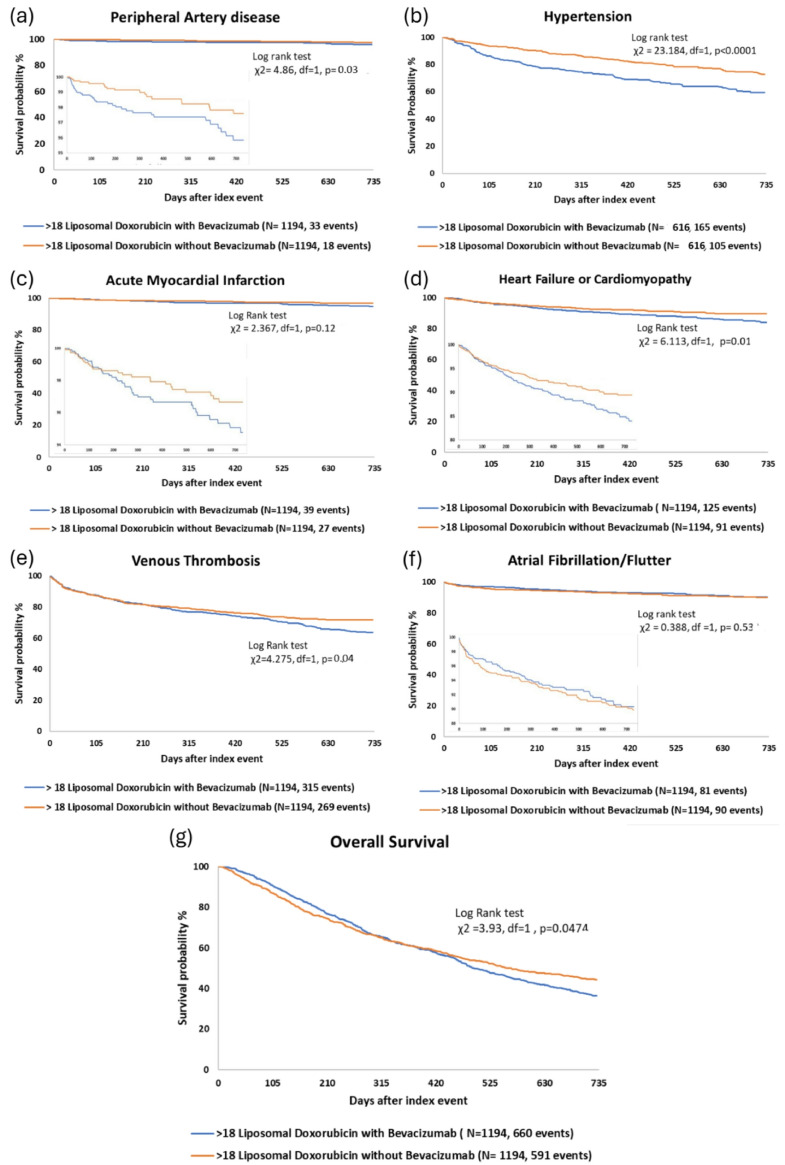
Kaplan-Meier survival curves comparing the risk of cardiovascular outcomes between propensity-matched cohorts from the TriNetX database receiving PLD with bevacizumab (blue) compared to PLD alone (orange). (**a**) Peripheral artery disease. (**b**) Hypertension, excluding 616 patients in each group with preexisting hypertension. (**c**) Acute myocardial infarction. (**d**) Cardiomyopathy or heart failure. (**e**) Venous thromboembolism. (**f**) Atrial fibrillation or flutter. (**g**) Overall survival

In a time to event analysis, the rates of congestive heart failure or cardiomyopathy (*p* = 0.0134, Figs. 2A-G), venous thrombosis (*p* = 0.0387), peripheral artery disease (*p* = 0.0275), and hypertension (*p* < 0.0001) were increased with combination therapy, while there was no significant difference in the rates of myocardial infarction or atrial fibrillation/flutter. The risk of all-cause mortality was increased in the cohort receiving combination therapy (*p* = 0.0474).

### Disproportionality analysis

**Fig. 3:**
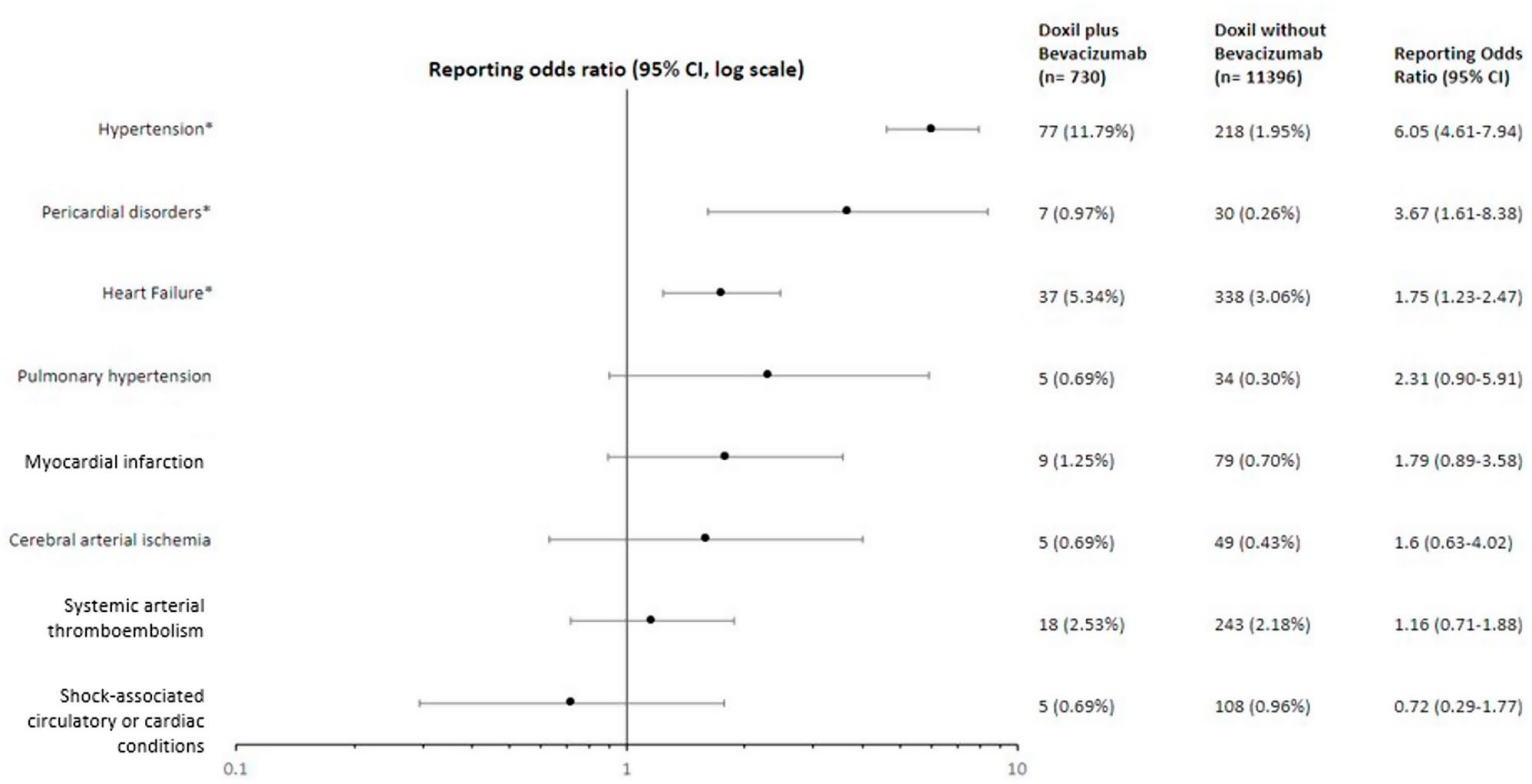
Disproportionality analysis of the risk of cardiovascular adverse event reporting for patients receiving PLD plus bevacizumab as compared to PLD alone as reported to the VigiBase database. All VigiBase cases reporting adverse events with PLD were extracted and pre-defined cardiovascular events were identified. Very rare events (defined as fewer than 5 cases reported) were excluded from analysis. Reporting odds ratios are given with 95% confidence intervals and are displayed on a logarithmic scale. The number and proportion of adverse event reports for each subtype of cardiovascular disease are as noted

A total of 11,396 case reports of adverse drug reactions associated with PLD only were identified through VigiBase, with 730 case reports of adverse drug reactions to PLD with bevacizumab. Combination therapy was associated with disproportionate reporting of hypertension (Fig. 3, 11.79% vs. 1.95%, ROR 6.05, 95% CI 4.61–7.94), pericardial disorders (defined in MedDRA as non-infectious pericarditis and pericardial disorders not otherwise classified; 0.97% vs. 0.26%, ROR 3.67, 95% CI 1.61–8.38), and heart failure (5.34% vs. 3.06%, ROR 1.75, 95% CI 1.23–2.47). There was no significant difference in the rates of reporting of myocardial infarction, pulmonary hypertension, diabetes, cerebral arterial ischemia, systemic arterial embolism, or cardiac death or shock. The rates of myocarditis, endocardial disorders, dyslipidemia, ventricular arrhythmia, supraventricular arrhythmia, Long QT/torsade de pointes, and cardiac conduction disorders were low in both groups and did not meet the frequency threshold for statistical analysis.

## Discussion

The principal findings of this study are that (i) combination therapy with PLD and bevacizumab is associated with increased risks of cardiotoxicity as compared to PLD therapy without bevacizumab in a propensity-matched cohort analysis, and (ii) combination therapy is associated with disproportionate reporting of cardiovascular adverse events to VigiBase, similarly indicating increased reporting risks of heart failure or cardiomyopathy as well as hypertension.

While prior studies have described increased rates of arterial thromboembolism, cardiac ischemia, and hypertension with angiogenesis inhibitors, including bevacizumab, our findings are notable for an increased risk of heart failure or cardiomyopathy with PLD co-treatment. In the AURELIA phase III trial of 361 subjects with platinum-resistant ovarian cancer randomized to chemotherapy (120 of whom received PLD) with or without the addition of bevacizumab, there were only two cases of heart failure and no other significant cardiovascular adverse events reported over a median of 13.9 months [7]. In contrast, a meta-analysis of 77 studies of angiogenesis inhibitors, including bevacizumab, in a variety of malignancies found a significant increase in the risk of cardiac dysfunction (incidence of 2.3%) with a non-significant trend towards an increased rate of heart failure with angiogenesis inhibitor treatment as compared to control. An increased risk of heart failure with bevacizumab treatment was demonstrated in another meta-analysis of bevacizumab when used for treatment of breast cancer [12, 13]. However, a significant proportion of breast cancer patients in these studies had prior exposure to other cardiotoxic therapies including anthracycline therapy, HER2-targeted therapies, or chest wall radiation. Another study describing patients undergoing bevacizumab for treatment for lung or colorectal cancer did not demonstrate an association between bevacizumab exposure and the development of heart failure [14]. Our findings add to this body of literature and suggest that in patients with gynecologic malignancies, PLD and bevacizumab may have a synergistic cardiotoxic effect.

The mechanisms by which angiogenesis inhibitors such as bevacizumab may contribute to cardiomyopathy have not been fully elucidated but may result from an increase in systemic blood pressures leading to myocardial hypertrophy and stunting of the myocardial microvasculature [15]. Bevacizumab has also been shown to be associated with heart failure when given in clinical trials with non-liposomal doxorubicin [3, 4]. Preclinical studies have described increased release of vascular endothelial growth factor-A (VEGF-A) from cardiac microvascular endothelial cells in response to doxorubicin exposure, a potentially cardioprotective response which is inhibited by exposure to VEGF-A antibodies such as bevacizumab [15, 16]. While PLD is associated with low rates of cardiotoxicity in isolation, co-administration with bevacizumab may alter innate pathways of cardioprotection and potentiate toxicity of this agent.

In this study, we observed an increased risk of all-cause mortality in patients receiving combination therapy in the TriNetX database. However, the exact cause of death and relative contributions of cancer versus cardiovascular disease cannot be ascertained in the TriNetX cohort. Our results are in contrast to those reported in the clinical trial setting, where a difference in the overall survival rate was not seen for combination chemotherapy plus bevacizumab versus chemotherapy alone [7]. It is conceivable that patients at a higher risk of cancer-related mortality are more likely to be treated with combination therapy in clinical practice. While the cohorts in this study were well-matched with regards to age, cardiovascular history and risk factors, cancer diagnosis, and the presence of metastatic disease, other unmeasured confounders may contribute to mortality in this patient population.

We also observed disproportionate reporting of pericardial disorders to VigiBase in patients treated with combination therapy. The factors contributing to this effect are unclear, as bevacizumab is not typically associated with pericarditis or pericardial effusions. One possible explanation is the presence of more advanced metastatic disease with pericardial involvement in patients selected for combination therapy. However, due to the limitations of disproportionality analysis, a causal relationship cannot be inferred based on AE reporting alone.

The strengths of this study include the use of two large observational patient cohorts, including a multicenter research network and a comprehensive international database of post-marketing drug adverse event reports, which were chosen as the creation of a prospective cohort of suitable size to detect cardiotoxicity of these agents was not feasible. Limitations of this study include the retrospective nature of the data as well as an inability to prove causality via disproportionality analysis. Additionally, cause of death is not able to be determined in the TriNetX cohort, which prevents accounting for the likely substantial competing risk of cancer-related mortality in this population. Translational studies are needed to further explore the mechanisms of cardiotoxicity of combined PLD with bevacizumab as well as to determine improved strategies for treatment and prevention.

## Conclusions

Combined PLD and bevacizumab therapy increases the risk of cardiotoxicity compared to PLD alone. These findings emphasize the need for proactive cardiovascular monitoring in patients undergoing this combination treatment. Prospective studies are warranted to further elucidate the underlying mechanisms and to refine clinical management strategies.

## Electronic supplementary material

Below is the link to the electronic supplementary material.

Supplementary Material 1

## Data Availability

The datasets used and/or analyzed during the current study are available from the corresponding author on reasonable request.

## Abbreviations

PLD: Pegylated Liposomal Doxorubicin
MACE: Major Adverse Cardiovascular Events
CV-ADR: Cardiovascular Adverse Drug Reaction
OR: Odds Ratio
CI: Confidence Interval
SD: Standard Deviation
VEGF: Vascular Endothelial Growth Factor
ROR: Reporting Odds Ratio
WHO: World Health Organization
MedDRA: Medical Dictionary for Regulatory Activities
PCI: Percutaneous Coronary Intervention
ACE: Angiotensin-Converting Enzyme
