# Supplementary material for: Cardiotoxicity of combined pegylated liposomal doxorubicin and bevacizumab therapy: a propensity-matched cohort study and disproportionality analysis
Source: Cardiooncology. 2025 Jul 2;11:59. doi: 10.1186/s40959-025-00351-4 (PMC12219240; doi:10.1186/s40959-025-00351-4)
Supplement: Supplementary file 1 — Supplementary Material 1 [file 40959_2025_351_MOESM1_ESM.docx]

**Supplemental Table 1: READUS-PV checklist**

| **Section and topic** | **Item #** | **Checklist item** | **Location where item is reported** |
| --- | --- | --- | --- |
| **Title** |  |  |  |
|  | *1a* | *If disproportionality analyses are a prominent component of the published study, the study should be identified as a “disproportionality analysis”. The type of data and name of the database(s) should be specified.* | *Title* |
|  | *1b* | *Report the name of adverse event(s) and/or drug(s) under study, when applicable.* | *Title* |
| **Introduction** |  |  |  |
| Background | *2a* | *Describe the drug(s) and its utilization, the nature of the adverse event(s) under study and its frequency, and the existing knowledge on the drug-event combination.* | *Background* |
|  | *2b* | *Specify the rationale for performing the analysis, e.g., as part of routine pharmacovigilance, to investigate an overall safety profile, or to assess a pre-specified hypothesis.* | *Background* |
|  | *2c* | *Explain why ICSR databases and disproportionality analysis are suitable to fill the knowledge gap.* | *Discussion* |
| Objectives | *3* | *State specific objectives, identifying the adverse event(s), the drug(s), and the reference group, including any pre-specified hypothesis, if applicable.* | *Background* |
| **Methods** |  |  |  |
| Study design | *4a* | *Identify the study (i.e., “disproportionality analysis”) and the type of data used (e.g., “individual case safety reports”).* | *Methods* |
|  | *4b* | *Provide an outline of the entire study design, including primary and sensitivity analyses performed, and other designs such as case-by-case analysis or literature review.* | *Methods* |
| Data description, access, and pre-processing | *5a* | *Specify the name of the database(s), the database(s) custodian, and the coverage. Specify the type/number of drugs included within the database and the thesaurus, taxonomies, or ontologies used for coding drugs and events.* | *Methods* |
|  | *5b* | *Specify the extraction dates and describe and justify all choices used for data pre-processing, including any data transformation or exclusion, if appropriate.* | *Methods* |
| Variables definition | *6a* | *Describe the study population, including any restriction.* | *Methods* |
|  | *6b* | *Describe the nature and the meaning of key variables assessed in the work.* | *Methods* |
|  | *6c* | *Specify and justify any grouping of drugs or events. For drugs, specify and justify whether active ingredients/trade names/salts were considered and/or the selected role.* | *Methods* |
|  | *6d* | *Describe any additional data source used, the type of data, and how they interact with ICSRs.* | *Methods* |
| Statistical methods | *7a* | *Present any descriptive analysis performed, specifying variables investigated, statistical tests, and significance thresholds.* | *Methods* |
|  | *7b* | *Describe the measure(s) selected for the disproportionality analysis including any threshold used to identify signals of disproportionate reporting. Explain the reason for this choice if applicable.* | *Methods* |
|  | *7c* | *Clearly describe any sensitivity analysis and any tool to control confounding, including any restriction, subgroup, stratification, adjustment, or interaction.* | *n/a* |
|  | *7d* | *Specify the variables and methods used for the case-by-case analysis, including any algorithm or criteria used to assess causality, if performed.* | *n/a* |
|  | *7e* | *Specify any statistical methods used for other data sources.* | *Methods* |
| **Results** |  |  |  |
| Participants | *8a* | *Specify the number of individual case safety reports included at each stage, including reasons for exclusion.* | *Results* |
|  | *8b* | *Provide key demographic and clinical characteristics of cases, if possible comparing cases with any appropriate reference group.* | *n/a* |
| Disproportionality analysis | *9* | *Present all results including confidence intervals. Present also results of sensitivity analyses, if performed.* | *Results* |
| Case-by-case analysis | *10* | *Present the case-by-case analysis of key variables. Present the causality assessment, if applicable.* | *n/a* |
| **Discussion** |  |  |  |
| Key results | *11* | *Discuss key results with reference to study objectives and contextualize them within the current literature and other consulted sources. Clearly discriminate between expected reactions and emerging safety signals.* | *Discussion* |
| External validity | *12a* | *Discuss the external validity of the results to the general population.* | *Discussion* |
|  | *12b* | *Discuss the potential relevance of results in clinical practice* | *Discussion* |
|  | *12c* | *Propose further study designs if applicable* | *Discussion* |
| Limitations | *13* | *Present general limitations, making clear that disproportionality analysis alone cannot prove causation or measure incidence, and specific limitations, including confounding and reporting bias and efforts to mitigate them.* | *Discussion* |
| **Declarations** |  |  |  |
|  | *14a* | *Provide the source of funding/sponsorship and the role of the funders/sponsors for the present study and for any original study on which the present article is based.* | *Declarations* |
|  | *14b* | *Clearly identify potential commercial and intellectual conflicts of interest (e.g., link to any drug/event investigated, whether financial, legal action, or software used).* | *n/a* |
|  | *14c* | *Declare any institutional approval needed or granted in the investigation.* | *Declarations* |
|  | *14d* | *Include a statement on data availability, code availability (including the version of the statistical software used), and protocol registration.* | *Declarations* |
